# Plasma glucose in screening for diabetes and pre-diabetes: how much is too much? Analysis of fasting plasma glucose and oral glucose tolerance test in Sri Lankans

**DOI:** 10.1186/s12902-019-0343-x

**Published:** 2019-01-22

**Authors:** G. W. Katulanda, P. Katulanda, C. Dematapitiya, H. A. Dissanayake, S. Wijeratne, M. H. R. Sheriff, D. R. Matthews

**Affiliations:** 1 Medical Research Institute, Colombo, 08 Sri Lanka; 2 Diabetes Research Unit, Department of Clinical Medicine, Faculty of Medicine, University of Colombo, Colombo, Sri Lanka; 3 Cruddas Link Fellow, Harris Manchester University, University of Oxford, Oxford, UK; 4 Oxford Centre for Diabetes Endocrinology and Metabolism, Oxford, London UK; 5 Faculty of Medicine, University of Colombo, Colombo, Sri Lanka

**Keywords:** Fasting plasma glucose, Diabetes, pre-diabetes, Sri Lanka, South Asia

## Abstract

**Background:**

Fasting plasma glucose (FPG) is the most commonly used screening tool for diabetes in Sri Lanka. Cut-off values from American Diabetes Association recommendations are adopted in the absence of local data. We aimed to establish FPG cut offs for Sri Lankans to screen for diabetes and pre-diabetes.

**Methods:**

Data on FPG and diabetes/pre-diabetes status were obtained from Sri Lanka Diabetes and Cardiovascular Study (SLDCS), a community based island wide observational study conducted in 2005–6. Sensitivity specificity and area under the ROC curve were calculated for different FPG values.

**Results:**

Study included 4014 community dwelling people after excluding people already on treatment for diabetes or pre-diabetes. Mean age was 45.3 (± 15) years and 60.4% were females. FPG cut off of 5.3 mmol/L showed better sensitivity and specificity than 5.6 mmol/L in detecting diabetes (87.8% and 84.4% Vs 80.8% and 92.1%) and pre-diabetes (54.7% and 87.0% Vs 43.8% and 94.2%).

**Conclusions:**

A lower FPG cut off of 5.3 mmol/L has a better sensitivity and acceptable specificity in screening for diabetes and pre-diabetes in Sri Lankan adults.

## Background

Diabetes mellitus is a growing health concern recognized as a pandemic by the American Diabetes Association (ADA) and has its 80% of affected population from the developing countries. For adults aged ≥ 20 years Sri Lanka has 10.3% (9.4–11.2%) overall prevalence of diabetes [males 9.8% (8.4–11.2%), females 10.9% (9.7–12.1%)] with a higher number of people with diabetes and pre-diabetes recorded form urban areas [1, 2]. But it is found that the number of people with diabetes identified in the rural areas of Sri Lanka has been progressively increasing with time [3] making diabetes no longer a disease of the rich.

Fasting Plasma Glucose (FPG) of ≥ 7.0 mmol/l, 2-h plasma glucose of ≥ 11.1 mmol/L after a standard 75 g Oral Glucose Tolerance Test (OGTT), HbA1c value of ≥ 6.5% and a casual blood glucose value of ≥ 11.1 mmol/L in a patient with classic symptoms of hyperglycemia are the criteria recommended by the ADA for diagnosis of diabetes mellitus [4]. Many now tend to use HbA1c as a screening test for diagnosis as it does not require patients to fast and reflects longer-term glycaemia than plasma glucose [5–7]. But in resource limited settings the lack of standardization, relative unavailability and high cost of the HbA1c assay promote FPG and OGTT as screening tests for diabetes among physicians.

OGTT was considered as the gold standard test for diagnosis of diabetes by the World Health Organization. But it is inconvenient for the patient, cumbersome for laboratory personnel and reproducibility is poor. Therefore FPG is the popular diagnostic tool for diagnosis of diabetes and pre-diabetes among physicians. However OGTT remains the diagnostic tool for diagnosis of diabetes during pregnancy.

The ADA recommends FPG for the estimation of incidence and prevalence of diabetes. However FPG fails to detect a population with diabetes who will be detected using WHO 1999 criteria (OGTT ≥ 11.1 mmol/L) [8, 9]. ADA defines pre-diabetes as either impaired fasting glucose (fasting plasma glucose between 5.6–6.9 mmol/L) or impaired glucose tolerance (2-h OGTT glucose 7.8–11.0 mmol/L).

The utility of OGTT and FPG in diagnosis of diabetes and pre-diabetes has not been assessed in Sri Lanka. OGTT was found to be more effective in diagnosing diabetes in American adults while FPG was found to be effective in those who are not willing to take OGTT [10]. The predictive ability of Impaired Fasting Glucose (IFG) on future diabetes risk was significantly low when compared to Impaired Glucose Tolerance (IGT) in some studies [11] while others have shown that future risk prediction of diabetes was similar for both IFG and IGT [2, 12].

There is a developing concern on modifying the cut-off values of FPG for diagnosing diabetes and pre-diabetes as it will increase the sensitivity for early detection [11, 13–15]. It was a necessity to develop cut off values specific to the region or country or to see the efficiency of ADA recommended cut-off values in our population as early detection and intervention will delay the progression of the disease.

## Methods

Sri Lanka Diabetes and Cardiovascular Study (SLDCS) was a cross sectional study conducted by the Diabetes Research Unit of the Faculty of Medicine, University of Colombo and the Oxford Centre for Diabetes Endocrinology and Metabolism of the University of Oxford UK. Ethical approval was obtained from the Ethical Review Committee of the Faculty of medicine, University of Colombo, and all participants provided informed written consent. Data collection was conducted between August 2005 and September 2006.

### Study population

SLDCS was carried out in seven out of all nine provinces in Sri Lanka; excluded were the North and the East provinces (14% of the total population) due to security. The total sample frame was approximately 14 million people living in 12,018 ‘village officer’ units. A multi-stage random cluster sampling technique was used to select a nationally representative sample of 5000 non-institutionalized adults ≥18 years of age. The sample size of each province and the rural and urban populations of each province were determined using a probability proportional to the size technique. The sample was recruited from 100 clusters having 50 households in each. A cluster size was determined as 50 households in order to achieve a satisfactory spread of all groups of the population based on the prior experience of the investigators. Clusters were selected by a computer generated random number list from the smallest governmental community administrative unit the ‘Village Office Units’. Voters’ lists were used to randomly select the first household of each cluster and a uniform criterion was used to select the remaining 49 households in all 100 clusters. The selected households were visited by the study team to provide information of the study and to randomly select one eligible adult ≥18 years from each household. Those who were pregnant, acutely ill or who declined participation were excluded.

### Data collection

Temporary data collection centers were established within or in close proximity to each cluster. The selected subjects were advised to visit the centers in the morning between 7.30 a.m. and 9.00 a.m. after an overnight fast of 10–12 h. They were advised to follow an unrestricted diet and to continue with usual physical activities for at least 3 days prior to the date of data collection. A trained interviewer administered, structured and pre-coded questionnaire was used for data collection. All participants underwent OGTT. Blood for glucose estimation were collected into sodium fluoride/potassium oxalate tubes, centrifuged shortly after collection at the data collection centre and plasma was separated. Plasma and serum were stored in ice boxes until they were frozen at − 20 °C within 6–12 h of collection. Biochemical tests were performed in a central laboratory in Colombo. Glucose assay was performed by an enzymatic (glucose oxidase) colorimetric method (Roche Diagnostics, Mannheim, Germany) in a Hitachi 704 chemical autoanalyser. The total coefficient of variation for glucose assay during the study period was 3.4%.

### Data analysis

Data was analysed using SPSS version17.0 (SPSS Inc. Released 2008. SPSS Statistics for Windows, Version 17.0. Chicago: SPSS Inc.). OGTT was considered as the reference standard for definition of diabetes and pre-diabetes, against which the FPG was compared to determine its diagnostic utility as a screening tool at population level.

## Results

Among the total of 4485 participants, 4014 were included in the analysis after excluding people already on treatment for diabetes (*n* = 343) and those with missing data (*n* = 128). The mean age of the population was 45.3 (± 15) years and 60.4% were females (Table 1).

**Table 1:** Population characteristics

Parameter	Number/value
Population analysed	4014
Age (years) (mean ± SD)	45.3 (± 15.0)
Females (%)	60.4%
BMI (kg/m^2^) (mean ± SD)	21.6 (± 4.2)
FPG (mmol/L) (mean ± SD)	4.85 (± 1.09)
2 h OGTT (mean ± SD)	6.14 (± 2.86)
Newly diagnosed diabetes (%)	191 (4.7)
Newly diagnosed IFG (%)	441 (10.9)
Newly diagnosed IGT (%)	548 (13.7)

*BMI* body mass index, *FPG* fasting plasma glucose, *IFG* impaired fasting glucose, *IGT* impaired glucose tolerance, *OGTT* oral glucose tolerance test

FPG between males [5.10 (± 1.02) mmol/L] and females [5.06 (± 1.08) mmo/L] did not show a significant difference but the 2 h post OGT glucose was higher in females [6.39 (± 2.81) mmol/L] compared to males [5.74 (± 2.90) mmol/L] (*p* <  0.05) (Table 2). In both men and women FPG and post OGT plasma glucose increased significantly with age. Highest FPG was seen in 50–59 age group in both men and women. Highest post OGT plasma glucose was seen in > 70 age group in both sexes. Men had significantly higher FPG compared to women in 20–29 and 30–39 age groups while women had significantly higher post OGT plasma glucose than men in < 19, 20–29, 30–39 and 40–49 age groups (Table 2).

**Table 2:** FPG and OGTT plasma glucose in men and women across age groups (*N* = 4014)

Age group	FPG (mmol/L)				2 h OGTT (mmol/L)			
	Men	Women	*P*	Total	Men	Women	*P*	Total
≤ 19	4.56	4.32	0.53	4.45	4.81	5.48	0.02	5.12
20–29	4.60	4.49	0.03	4.54	4.93	5.63	< 0.01	5.34
30–39	4.95	4.72	0.02	4.80	5.39	6.08	< 0.01	5.82
40–49	5.21	5.18	0.75	5.19	5.82	6.70	< 0.01	6.36
50–59	5.39	5.49	0.44	5.45	6.17	6.65	0.03	6.50
60–69	5.32	5.32	0.99	5.32	6.20	6.69	0.07	6.47
≥ 70	5.15	5.35	0.52	5.26	6.74	7.06	0.17	6.88
Total	5.10	5.06	0.51	5.08	5.74	6.39	< 0.01	6.14

*2 h OGTT* 2-h oral glucose tolerance test, *FPG* fasting plasma glucose

**Fig. 1:**
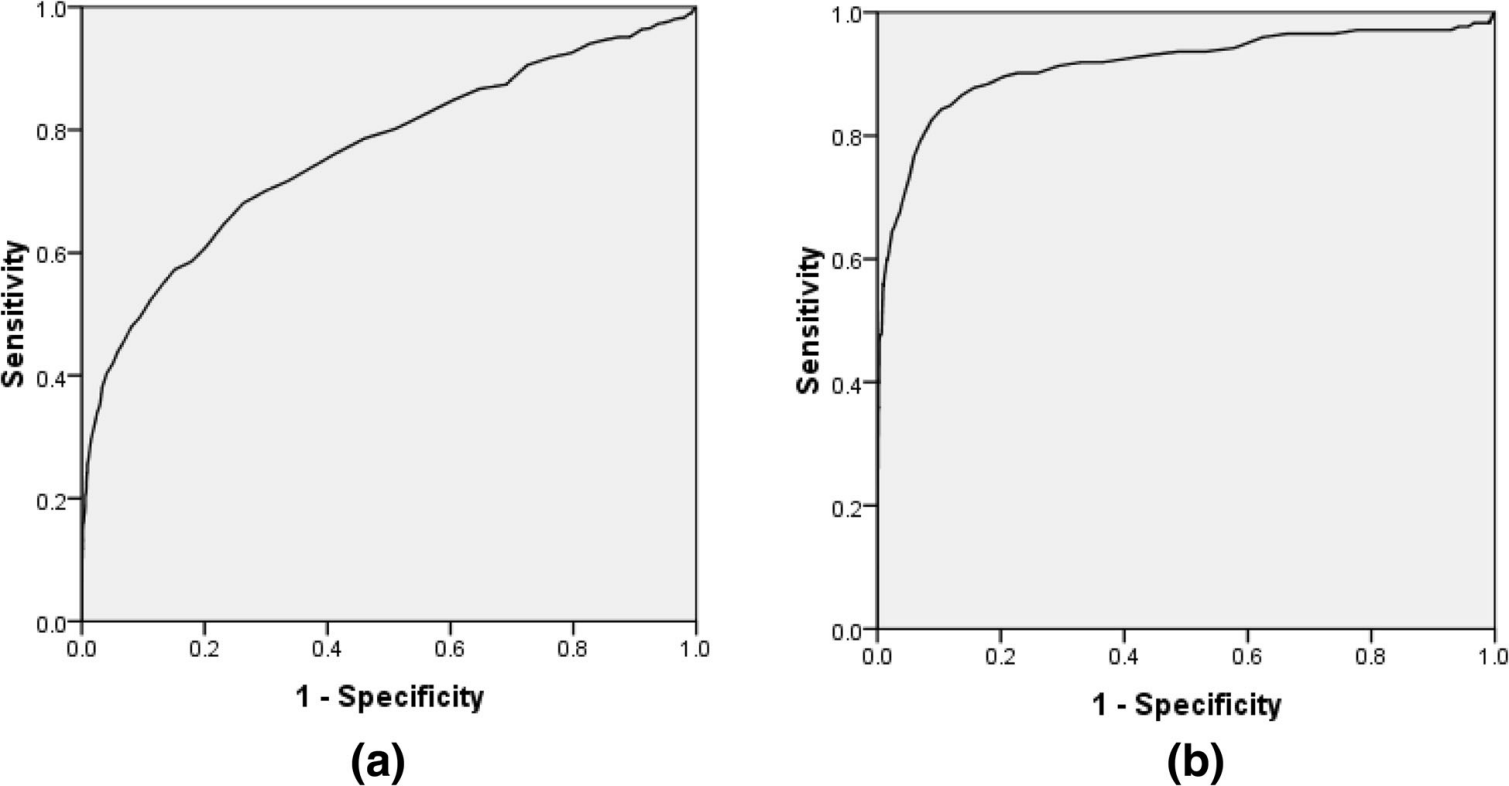
ROC curve for FPG in screening (**a**) for pre-diabetes/diabetes; (**b**) for diabetes

Area under the ROC curve was 0.76 for FPG in diagnosing diabetes while it was 0.91 for diagnosing pre-diabetes or diabetes (Fig. 1).

The sensitivity of FPG to diagnose pre-diabetes or diabetes is 43.8% when the cut off is 5.6 mmol/L with a specificity of 94.2% (Graph 1). The sensitivity to identify diabetes mellitus is 80.8% with a specificity of 92.1% at the same cut off value (Table 3). An FPG cut off value of 5.3 mmol/L has noticeably higher sensitivity with reasonable specificity for diagnosis of pre-diabetes / diabetes (Table 3).

**Table 3:** Sensitivity and specificity for FPG in diagnosing both pre-diabetes/diabetes and diabetes

FPG (mmol/L)	Pre DM and DM		DM only	
FPG cut off	Sensitivity	Specificity	Sensitivity	Specificity
4.4	87.4	30.9	96.5	29.5
5.0	68.1	73.7	91.3	70.7
5.3	54.7	87.0	87.8	84.4
5.6	43.8	94.2	80.8	92.1
6.1	26.6	98.9	62.2	98.0
7.0	15.8	99.8	47.1	99.6

## Discussion

SLDCS is the largest population based study on diabetes conducted in Sri Lanka.

### Patterns of FPG and OGTT results

Our female population had a higher overall post OGTT plasma glucose levels compared to males (*p* <  0.05). Relatively poor glucose handling was also seen in women in several other studies [16–20] but all involved women only in the middle age to elderly. Reason for this observation is uncertain. Longer gut half-life of glucose in women and inverse relationship between glucose tolerance and body height may at least partly explain this observation [16, 19]. Higher rates of IGT among women and rising OGT glucose values with age may contribute to higher risk of macro and microvascular complications of diabetes in this population. Currently practiced 2 h OGTT values for diagnosis of pre-diabetes and diabetes showed good sensitivity and specificity.

FPG was higher among men compared to women although the difference was statistically insignificant. Similar observations have been made in other studies [16–20]. Though the FPG reached a peak at 50–59 years, OGTT increased with aging. In contrast, studies in other countries have shown a significant increase in both FPG and post OGTT plasma glucose levels with aging [21–23] which is thought to be occurring partly due to reduced muscle mass and physical inactivity. This raises the need for the addition of post OGTT plasma glucose level for increasing the sensitivity for detection of diabetes in the elderly in our population.

### FPG cut off for pre-diabetes/diabetes screening

According to our analysis, an FPG cut off value of 5.6 mmol/L had a good specificity (94.2%) for diagnosis of abnormal hyperglycemia (pre-diabetes and/or diabetes) but had a relatively poor sensitivity (43.8%). But for diagnosis of diabetes alone it had higher sensitivity (80.8%) and specificity (92.1%). Once the cut off value was lowered furthermore it improved the sensitivity at the expense of minor lowering of specificity. We identified an FPG level of 5.3 mmol/L as the most appropriate cut off value for our population in diagnosing both pre-diabetes and/or diabetes.

Lower cut off values for FPG were also recommended by other Asian countries ranging from 5.6–6.3 mmol/L for diagnosis of diabetes [13–15, 24]. However in contrast to our results for diagnosis of pre-diabetes, several other studies recommend a cut off value of 5.6 mmol/L as a good cut off with a sensitivity and specificity close to 100% [25, 26]. Nevertheless none of the above studies involved South Asian population and in the majority participants were > 35 years of age. What caused these differences in FPG levels in the Sri Lankan population is unclear.

We had several limitations in our study. First, only a single FPG or OGTT was used for defining the cases in our study as pre-diabetes or diabetes and a repeated confirmation test was not carried out. But we assume the impact of this to be very small due to the large number of participants. Second, the study did not include participants from North and East provinces. Data from more nationally representative sample as well as from other countries of the region will give more robust information about optimum cut points. Third, our study sample comprised of more women than men. However, no significant difference was observed in cut points for men and women (data not shown).

## Conclusions

In resource limited settings, FPG and OGTT are valuable tests in diagnosing diabetes and pre- diabetes. However, ethnic and genetic variations in metabolism would suggest that single cut off may not be applicable to all universally. Fasting plasma glucose cut off of 5.3 mmol/L has a better sensitivity and acceptable specificity for diagnosis of pre-diabetes and diabetes among Sri Lankan adults. This will allow early identification of individuals at risk of progressing to diabetes and early targeted life style interventions to prevent development of diabetes and its complications. In addition, Sri Lankan female population has a relatively poor glucose handling compared to males and is more prominent in the reproductive age group. OGT also deteriorates with age than FPG. Therefore we suggest that performing OGTT in elderly would improve diagnostic rates of diabetes/pre-diabetes.

## Abbreviations

ADA: American Diabetes Association
FPG: Fasting Plasma Glucose
HbA1c: Glycated haemoglobin A1
IFG: Impaired fasting glycaemia
IGT: Impaired glucose tolerance
OGT: Oral glucose tolerance
OGTT: Oral glucose tolerance test
SLDCS: Sri Lanka Diabetes and Cardiovascular Study
WHO: World Health Organization
